# Poly(imide)/Organically-Modified Montmorillonite Nanocomposite as a Potential Membrane for Alkaline Fuel Cells

**DOI:** 10.3390/membranes2030430

**Published:** 2012-07-18

**Authors:** Liliane C. Battirola, Luiz H. S. Gasparotto, Ubirajara P. Rodrigues-Filho, Germano Tremiliosi-Filho

**Affiliations:** Instituto de Química de São Carlos, Universidade de São Paulo, São Carlos 13560-970, São Paulo, Brazil; Email: lcbattirola@iqsc.usp.br (L.C.B.); uprf@iqsc.usp.br (U.P.R.F.); germano@iqsc.usp.br (G.T.F.)

**Keywords:** clay-modified poly(imide), alkaline fuel cell, membranes

## Abstract

In this work we evaluated the potentiality of a poly(imide) (PI)/organically-modified montmorillonite (O-MMT) nanocomposite membrane for the use in alkaline fuel cells. Both X-ray diffraction and scanning electron microscopy revealed a good dispersion of O-MMT into the PI matrix and preservation of the O-MMT layered structure. When compared to the pure PI, the addition of O-MMT improved thermal stability and markedly increased the capability of absorbing electrolyte and ionic conductivity of the composite. The results show that the PI/O-MMT nanocomposite is a promising candidate for alkaline fuel cell applications.

## 1. Introduction

Alkaline fuel cells (AFCs) are the oldest and most matured among the distinct types of fuel cells that use concentrated potassium hydroxide as liquid electrolyte [1]. They were first introduced by Reid [2] and have been under development for space programs since the 1950s. However, other fuel cell technologies have emerged in the scenario such as the proton-exchange membrane fuel cell (PEMFC), mostly due to the possibility of employing a solid electrolyte and the avoidance of leakage of the highly alkaline electrolyte. Besides, the efficiency of the AFCs decreases because of the carbonatation reaction [3]. Carbonates would precipitate blocking the electrolyte flow and electrode pores on Pt-based electrodes. In addition, the carbonate crystals can also mechanically deteriorate the active layer [4].

In order to solve these difficulties, solid polymer electrolyte (SPE) membranes have been proposed for the use in AFCs [5]. The alkaline anionic exchange membranes (AAEMs) may offer a solution concerning the alkaline medium [6]. The lack of mobile cations avoids the precipitation of metal carbonate solid crystals. A further advantage is the potential use of non-noble metals [7] due to the low overpotentials associated with the electrochemical reactions at high pH. Candidates for anionic membranes have to efficiently transfer hydroxyl ions from one electrode to other, must be a barrier to hydrogen and oxygen (or to other fuels) and have to be chemically and mechanically stable [8]. In this context, polymer/layered silicates have attracted considerable interest from both industry and academia due to their remarkably enhanced properties when compared to virgin polymers [9]. The improvements comprise high moduli [10], increased strength and heat resistance [11], improved gas impermeability [12] and inflammability [13]. Jung *et al.* [14] produced Nafion^®^/montmorillonite (MMT) nanocomposite membranes for direct methanol fuel cell. They found the permeability of the composite membrane to decrease with increasing contents of MMT. Furthermore, when compared to the pure membrane, the Nafion^®^/MMT composite improved the performance of the membrane-electrolyte assembly at high operating temperatures. Chuang *et al.* [15] evaluated a nanocomposite membrane consisting of polybenzimidazole (PBI) and an organically-modified MMT clay for direct methanol fuel cell applications. The authors found that the mechanical properties and methanol barrier ability of the PBI films were substantially enhanced upon incorporation of MMT. 

Given that the addition of clays may enhance the performance of membranes for fuel cell purposes, in this work we synthesized a MMT-modified poly(imide) nanocomposite and evaluated its potentiality as a membrane for AFC applications. Poly(imide) is known for its thermal stability, excellent mechanical properties and good chemical resistance, therefore a promising candidate for fuel cell applications. The thermal stability and physical properties of the nanocomposite membrane were characterized with Fourier transform infrared spectroscopy (FTIR), thermal gravimetric analysis (TGA), scanning electron microscopy (SEM), and X-ray diffraction (XRD). We also discuss the effect of temperature on the ionic conductivity of the nanocomposite membrane.

## 2. Results and Discussion

**Figure 1:**
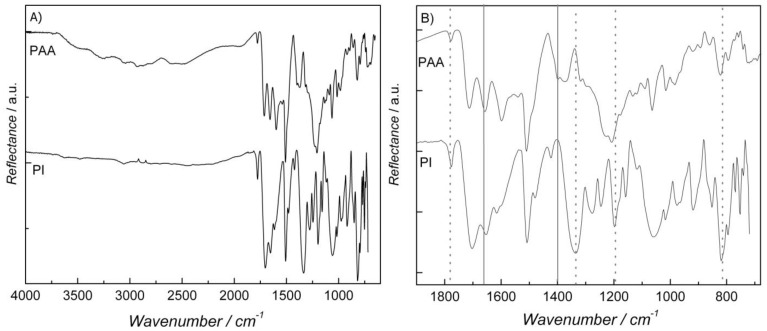
Fourier transform infrared spectroscopy (FTIR) spectra of pure pre-polymer (PAA) and poly(imide) (PI). (**A**) Full-range spectra and (**B**) expended low-frequency region. Vertical full and dotted lines in (**B**) indicate bands that vanished and appeared, respectively, as a consequence of the polymerization process.

**Figure 2:**
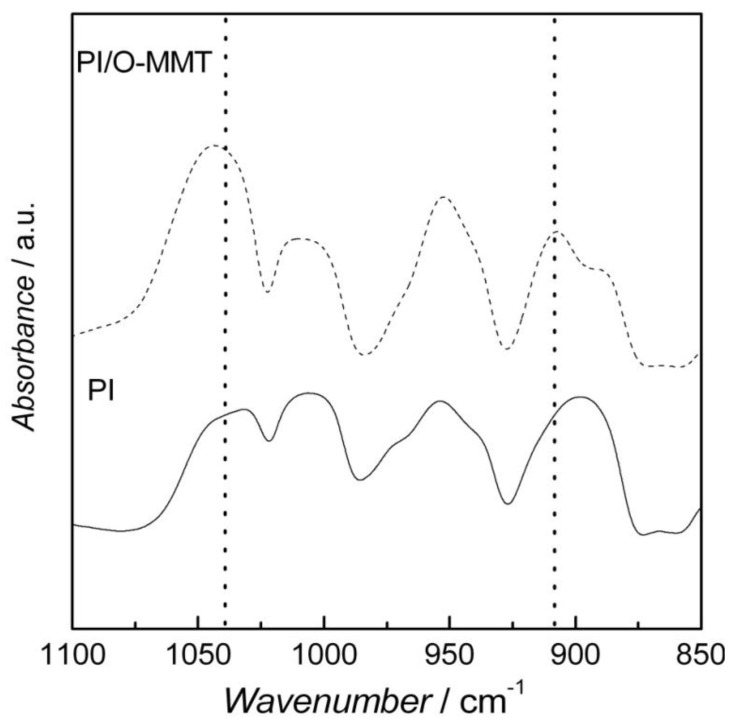
FTIR spectra of pure PI (full line) and PI/organically-modified montmorillonite (O-MMT) (dashed line).

A two-step method was used to synthesize the PI. BTDA was mixed with *p*-FDA in DMAc to form the pre-polymer (PAA). PAA was then subjected to a heating program and its conversion into PI can be observed in the FTIR spectra of Figure 1. Bands between 2,500 cm^−1^ and 3,300 cm^−1^ for PAA (Figure 1A) ascribed to O–H and N–H stretchings disappear in the PI spectrum, which evidences the formation of the imide ring. Further evidence of the imide ring formation can be observed in the expanded low-frequency region in Figure 1B. Bands at around 1,405 cm^−1^ and at 1,660 cm^−1^ (full vertical lines) due to the stretching of C–N and C=O of primary amides are no longer observable in the PI spectrum, which suggests the PAA condensation into PI. Moreover, the appearance of bands at 1,780 cm^−1^ (assimetric C=O stretching), 1,340 cm^−1^ and 1,205 cm^−1^ (C–N stretching), and around 800 cm^−1^ (N–H vibration) is related to the imide ring. The above-ascribed frequencies suggest a cis-trans isomerism, in which one carbonyl group is *cis* and the other *trans* to the N–H group [16]. The PI/O-MMT nanocomposite was also analyzed with FTIR and the results are depicted in Figure 2. Characteristic absorption bands of O-MMT at 1,040 cm^−1^ and 917 cm^−1 ^due to Si–O–Si and Al–O–Al, respectively, confirm the incorporation of O-MMT into the PI matrix. 

**Figure 3:**
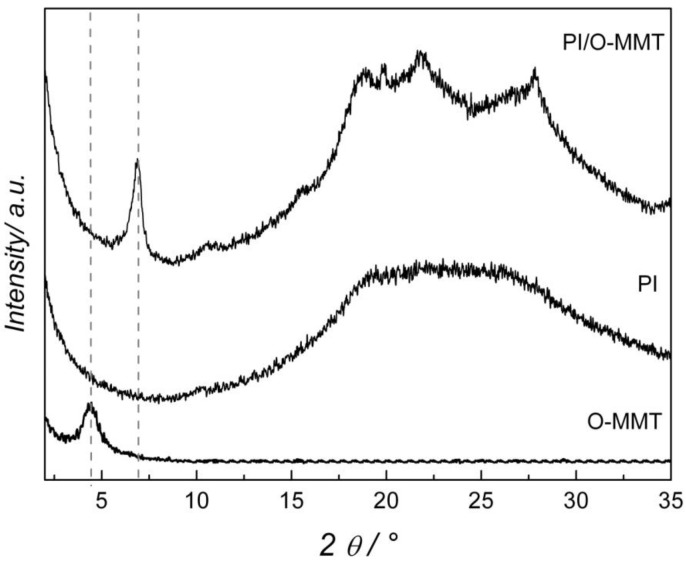
X-ray diffraction patterns of pure O-MMT, pure PI and PI/O-MMT.

The intercalation of PI in the interlayers of O-MMT is evidenced by the XRD patterns shown in Figure 3. The pure PI diffractogram presents only a broad region (between 15° and 30°) characteristic of a non-crystalline material. The XRD pattern of the pure clay gives a peak at 4.7° due to the (001) plane (*d*-spacing of 18.8 Å). Upon adding the O-MMT to the PI, the value of the (001) plane is shifted to 6.9°, implying a *d*-spacing of 14.3 Å. The decrease of the *d*-spacing suggests that the quaternary ammonium salts of the O-MMT are replaced by the PAA in the interlayer region with its subsequent polymerization into PI. This unexpected result (the *d*-spacing decrease) was also observed by Lan *et al.* [17] who suggested that the PI acquires a more compact conformation in the clay interlayer region. Moreover, the appearance of peaks between 15° and 30° indicates an increase in the crystallinity of the sample, suggesting that the interlayer region of the O-MMT also functioned as a site for nucleation and growth of the polymer.

**Figure 4:**
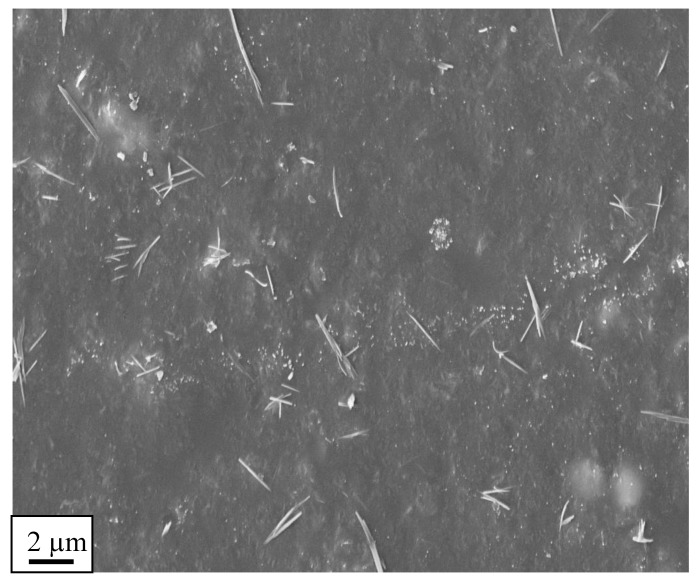
Scanning electron microscopy (SEM) micrograph of the PI/O-MMT nanocomposite.

SEM micrograph of PI/O-MMT nanocomposite is shown in Figure 4. The dark-grey areas in the figure are the PI matrix and the white parts are the O-MMT silicate layers. The randomly-dispersed needle-like silicate structures are estimated to be ~110 nm in thickness and 0.7–4 µm in length. Although some exfoliation may have occurred (white-blurred regions in the micrograph), the layered structure of the clay was preserved, a result corroborated by the XRD data. The clay is well dispersed in the PI matrix without preferential orientation.

**Figure 5:**
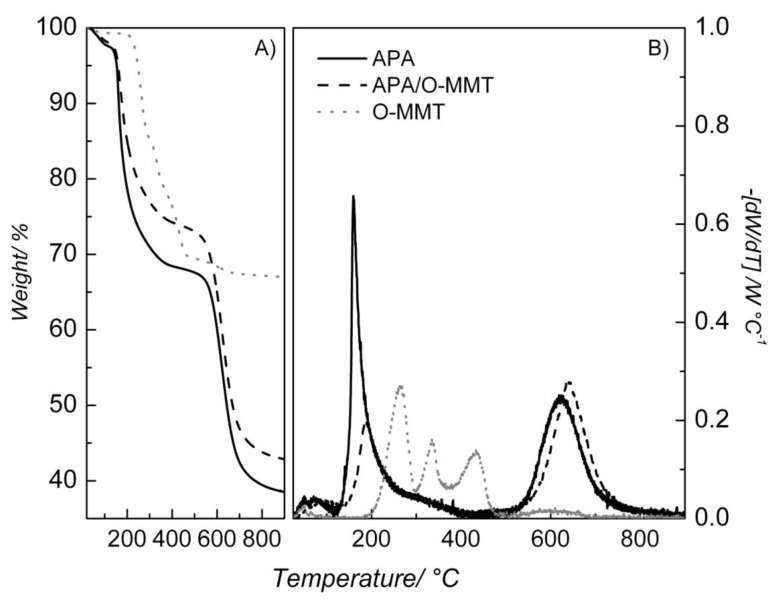
Thermal gravimetric analysis (TGA) (**A**) and DTGA (**B**) curves for the materials employed in this work. The identity of the samples is indicated in the figures.

The thermal stabilities of O-MMT, PAA/O-MMT and pure PAA were examined with TGA. There are three weight-loss regions in Figure 5A. For the experiments the pre-polymer (APA) was used since it becomes PAA upon heating. The first region (from room temperature to around 110 °C) is related to evaporation of DMAc and water. The second event (from 250 °C to 480 °C) is ascribed to the loss of oligomers, CO, CO_2_ and water [18]. At the highest temperatures, 500–850 °C, degradation of the samples take place. The enhanced thermal stability of the nanocomposite is more clearly observed in the DTGA curves of Figure 5B. The temperature values of the third event for the PAA were shifted to higher ones in the case of the PAA/O-MMT, attesting an improvement of the thermal resistance of the latter material. The preserved layered structure of the clay (Figure 4) may have shielded the PAA from the heat delaying the thermal decomposition of the system [19]. Although the nanocomposite is stable up to 500 °C, for a real fuel cell application the operating temperature should be at around 200 °C. Temperatures higher than the latter could lead to thermal decomposition of the clay surfactant, a species also responsible for the ionic conductivity of the nanocomposite. 

**Figure 6:**
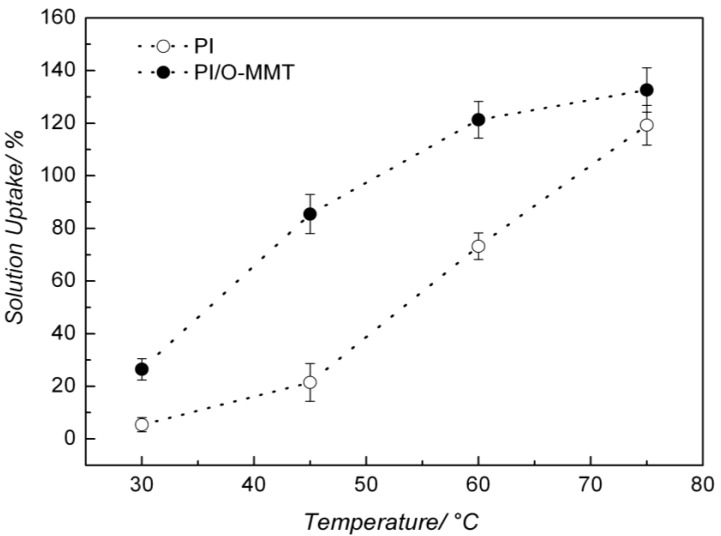
Aqueous 1.5 mol L^−1^ KOH uptake by PI and PI/O-MMT nanocomposite.

One of the factors that regulate the fuel cell efficiency is the ability of the membrane in absorbing the electrolyte. Figure 6 shows the 1.5 mol L^−1^ KOH uptake by the PI/O-MMT nanocomposite at distinct temperatures. Results for the unmodified PI are also presented for comparison. One can see an increase of the KOH solution uptake with the increasing temperature for both materials. However, in the case of the PI/O-MMT, the values of solution absorption are higher than those for the pure PI. This indicates that the clay increased the porosity of the PI. This behavior can be clearly seen in Table 1, where weight increments of 490%, 390%, 65% and 11% at 30 °C, 45 °C, 60 °C and 75 °C, respectively, where observed upon addition of O-MMT to PI. 

**Table 1:** Values of solution uptake by PI and PI/O-MMT at different temperatures.

Temperature (°C)	Solution Uptake (%)		Increment (%)
	PI	PI/O-MMT	
30	5.40 ± 2.70	26.46 ± 4.02	~390
45	21.49 ± 7.20	85.47 ± 7.42	~298
60	73.25 ± 5.05	121.33 ± 6.97	~66
75	119.25 ± 7.58	132.63 ± 8.40	~11

**Figure 7:**
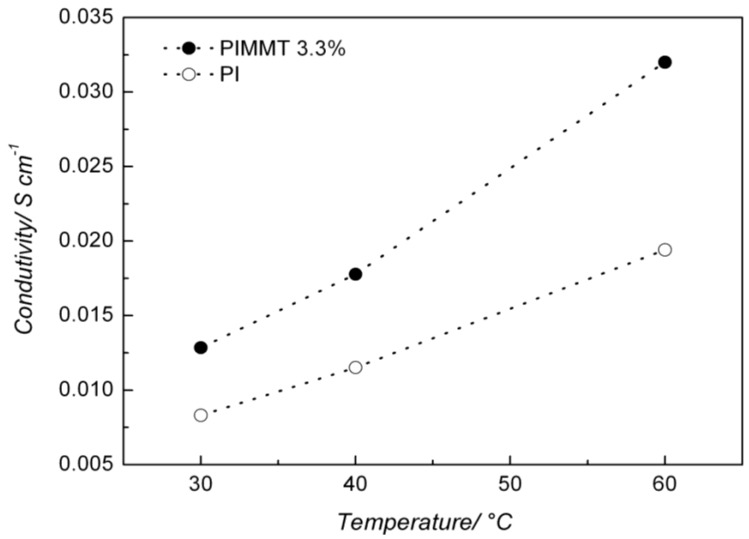
Conductivity for pure PI and PI/-O-MMT nanocomposite as a function of temperature.

Figure 7 depicts the ionic conductivities of the PI and PI/O-MMT nanocomposite as a function of temperature. Both membranes exhibited a marked increase in conductivity with increasing temperature. This is due to the role played by temperature in the kinetics of anion mobility in the polymeric membrane as well as in the motion of polymer chains [14]. Table 2 presents values of conductivities for both materials at different temperatures. One can see that the PI/O-MMT nanocomposite exhibited higher conductivity values than those of pure PI at all investigated temperatures, a result which attests the improved performance of the modified PI. As previously mentioned, the clay may have increased the porosity of the membrane, therefore contributed to increasing the nanocomposite conductivity. The material’s conductivity could be explained by Grotthuss-like [20] mechanism as well as the formation of pores, probably interconnected, that increase the ionic diffusivity. Furthermore, the presence of the surfactant may also play a role in increasing the conductivity through *surface site hopping*, since the XRD showed the surfactant was expelled from the organoclay’s interlayer. We should mention that the membrane is not perfectly selective, and potassium ions may accumulate at the air-electrode interface with precipitation of potassium hydrogen carbonate. The conductivities obtained in this work are still lower than those observed for Nafion^®^ [21,22] (around 0.1 S cm^−1^) in the same temperature range. However, compared to the conductivities of polybenzimidazole (PBI)/MMT (around 0.00001 S cm^−1^) nanocomposite [15] measured at 160 °C, the PI/O-MMT reported in this work exhibited conductivities at least one hundred times higher at a much lower temperature (60 °C). 

**Table 2:** Values of conductivities at the investigated temperatures.

Temperature (°C)	Conductivity (S cm^−1^)		Increment (%)
	PI	PI/O-MMT	
30	0.008	0.013	62
40	0.011	0.018	63
60	0.019	0.032	68

## 3. Experimental Section

### 3.1. Chemicals

All reagents were used without prior purification. Solutions were prepared with ultrapure Milli-Q water (18.2 Ω cm^−1^). 97% 1,4-phenylene diamine (*p*-FDA), 96% 3,3',4,4'-dianhydride tetracarboxyl benzophenone (BTDA), and 99% *N,N'*-dimethylacetamide (DMAc) were purchased from Aldrich. Organo Modified Clay (Montimorilonite (O-MMT)) was kindly supplied by Professor Gournis, University of Ioannina, and its synthesis procedure is described elsewhere [23]. Briefly, a solution of the surfactant hexadecyl-trimethyl ammonium bromide in water (1.5 times the CEC of the clay) was slowly added to an aqueous 1 wt % clay suspension under vigorous stirring. The mixture was stirred for 24 h, centrifuged, washed with water three times, and air-dried by being spread on a glass-plate. 

### 3.2. Polymer Synthesis and Modification with O-MMT

The synthesis of the pure poly(imide) (PI) was carried out by adding 1.6 mmol of BTDA to a solution of 1.6 mmol *p*-FDA in 7 mL of DMAc. The mixture was stirred for 2 h at room temperature yielding a viscous 10 wt % poly(amic acid) (PAA) solution. Afterwards, 5 mL of DMAc was added to the PAA and the final mixture was then casted in a Teflon mold and kept overnight at 80 °C to evaporate most of the solvent. In order to generate the PI, the semi-dried PAA was subjected to the following heating program: 100 °C/30 min, 200 °C/30 min and 300 °C/30 min under inert atmosphere. 

The modification of PI with O-MMT was conducted as follows: A suspension of 0.25 g of O-MMT in 25 mL of DMAc was sonicated for 15 min, followed by reflux at 150 °C for 24 h. A determined volume of the suspension (3.3 wt % related to PI weight) was added together with 1.6 mmol BTDA into the 1.6 mmol *p*-FDA solution. From this point the procedure for obtaining the PI/O-MMT nanocomposite is the same of that for the pure PI.

### 3.3. Characterization

FTIR spectra were acquired with an IFS 66 Bruker spectrometer in the attenuated total reflectance (ATR) mode with a 4 cm^−1^ spectral resolution and average of 100 scans. The optical path was purged with nitrogen to eliminate peaks of water vapor and carbon dioxide. X-ray diffraction experiments were conducted on a Rigaku Ultima IV diffractometer with Cu *K_α_* radiation (*λ* = 1.54056 Å) at 40 kV and 40 mA. PI/O-MMT membranes were examined by scanning electron microscopy (SEM) using a Zeiss-Leica/LEO 440 model (LEO, UK). Prior SEM analyses all composite films were covered with a 10 nm gold layer to promote electron conductivity. The thermal stability of the composite membrane was investigated with a Perkin Elmer Thermal Analysis equipment. 5 mg of PAA with and without 3.3 wt % of MMT were placed into an alumina sample holder and subjected to heating from 25 °C to 900 °C at a heating rate of 10 °C min^−1^ under N_2_ flow of 20 mL min^−1^.

Measurements of electrolyte uptake were conducted by keeping the dried membrane samples immersed in a 1.5 mol L^−1^ KOH solution for four hours at different temperatures (30 °C, 45 °C, 60 °C and 75 °C). The weights of the swollen membranes were then measured after removing the excess of liquid with a soft paper and the solution uptake was determined with the following equation:

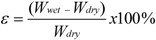

where *ε* is the solution uptake and *W_wet_* and *W_dry_* are the wet and dry membrane weights, respectively.

The ionic conductivities of the hybrid composite membranes were measured with an AutoLab 302N potentiostat/galvanostat in a fuel cell setup [24,25]. The membranes were manually pressed (contact area of 0.5 cm^−2^) between two carbon-supported Pt-nanoparticle electrodes (30 wt % Pt/C) and heating plates were housed in the system to allow temperature modulation from 25 °C to 60 °C. Impedance measurements were conducted from 100 kHz to 1 Hz with a voltage amplitude of 10 mV. The H_2_ pressure applied on both sides of the probe cell was 10 mbar. The values of ionic conductivities were calculated with the following equation:

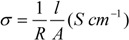

where *σ*, *R*, *l* and *A* are the ionic conductivity, resistance, thickness, and area of the samples, respectively. The resistance is directly measured through impedance and then inserted into the equation to obtain the conductivity.

## 4. Conclusions

The O-MMT was successfully incorporated into PI. XRD data revealed an intercalation of PI in the interlayers of O-MMT. The addition of the organically modified clay into the poly(imide) improved the properties of the polymer for a potential application in alkaline fuel cells. The PI/O-MMT nanocomposite exhibited higher thermal stability and ionic conductivity than the pure PI, prerequisite parameters for AFCs.
